# Effect of Systemic Calcitonin Delivery with and Without Adjunct Local Platelet-rich Fibrin Therapy on Osseointegration in Ovariectomized Osteoporotic Rabbits: A Scanning Electron Microscopy-based Study

**DOI:** 10.3290/j.ohpd.b1693857

**Published:** 2021-07-15

**Authors:** Maha G. Omar, Sahar Mahmoud El-Refai, Eman Khalil, Munerah Saleh BinShabaib, Shatha Subhi AlHarthi

**Affiliations:** a Professor, Department of Oral Medicine, Diagnosis and Periodontology, College of Dentistry, Princess Nourah Bint Abdulrahman University, Riyadh, Saudi Arabia. Study design, statistical analysis.; b Assistant Professor, Department of Oral Diagnostic Sciences, Oral Pathology, College of Dentistry, Princess Nourah Bint Abdulrahman University, Riyadh, Saudi Arabia. Study design, statistical analysis.; c Associate Professor, Department of Oral Medicine, Diagnosis and Periodontology, Faculty of Dentistry, British University of Egypt, Cairo, Egypt. Animal care, histology.; d Assistant Professor Periodontology, Department of Preventive Dental Sciences, College of Dentistry, Princess Nourah Bint Abdulrahman University, Riyadh, Saudi Arabia. Study design, wrote manuscript.; e Assistant Professor Periodontology, Department of Preventive Dental Sciences, College of Dentistry, Princess Nourah Bint Abdulrahman University, Riyadh, Saudi Arabia. Histology, wrote manuscript.

**Keywords:** calcitonin, gap junctions, osseointegration, osteoporosis, platelet rich fibrin.

## Abstract

**Purpose:**

It is hypothesised that systemic calcitonin delivery with adjunct local platelet-rich fibrin (PRF) therapy is more effective in augmenting osseointegration than calcitonin delivery alone under experimental osteoporosis conditions. The primary objective of the present experiment was to assess the effect of systemic calcitonin delivery with and without adjunct local PRF therapy on osseointegration in ovariectomised osteoporotic rabbits.

**Materials and Methods:**

Thirty female bilaterally ovariectomized rabbits were used. The animals were fed a low-calcium diet to establish a model for osteoporosis. In each animal, 2 implants were bilaterally placed in tibia. The animals were randomly divided equally into 3 groups. In group 1, no treatment was offered (control group). In groups 2 and 3, the animals received intramuscular injections of calcitonin without and with local PRF delivery prior to implant placement, respectively. All animals were euthanised at 12 weeks, and osseointegration was assessed as the gap widths between the bone and implant surface in the cervical, middle and apical third using scanning electron microscopy and energy-dispersive x-ray spectroscopy. The bone-to-implant contact (BIC) was also measured. p < 0.05 was defined as statistically significant.

**Results:**

Gap widths in the cervical (p < 0.001), middle (p < 0.001) and apical third (p < 0.001) were statistically significantly higher in group 1 than groups 2 and 3. Gap widths in the cervical (p < 0.001), middle (p < 0.001) and apical third (p < 0.001) were significantly higher in group 3 than group 2. The mean BIC was statistically significantly higher in the cervical (p < 0.001), middle (p < 0.001) and apical third (p < 0.001) in group 3 compared with groups 2 and 3.

**Conclusions:**

When used as an adjunct to calcitonin, PRF enhanced osseointegration in an experimental osteoporosis model. However, further well-designed studies with inclusion of additional groups (treatment with PRF alone) are needed.

Dental implants are a state-of-the-art technology used for the oral rehabilitation of partially and completely edentulous patients. Factors such as attainment of primary stability and direct bond between bone and implant body (osseointegration) play an essential role in this regard.^17,20^ However, a dilemma in clinical implant dentistry is the occurrence and progression of peri-implant diseases (peri-implant mucositis and peri-implantitis).^27^

Osseointegration was originally defined as a direct structural and functional connection between ordered living bone and the surface of a load-carrying implant.^3^ Today, an implant is regarded as osseointegrated when there is no progressive relative movement between the implant and the bone with which it is in direct contact. Systemic risk factors that have been linked with loss of osseointegration, which may lead to implant loss, include a state of chronic hyperglycemia and a compromised bone mineral density (BMD), which are common manifestations in patients with poorly-controlled diabetes mellitus (DM) and osteoporosis, respectively.^11,21^ Osteoporosis is a skeletal disorder characterised by low BMD and volume (type-IV bone).^30^ According to the International Osteoporosis Foundation, osteoporosis affects nearly 300 million individuals worldwide and is more common among older females.^33^ It has been reported that impaired bone metabolism complicates the maintenance of osseointegration by jeopardising the bone-to-implant contact (BIC), thereby increasing the risk of implant loss .

Calcitonin is a naturally-occurring peptide that minimises bone resorption by inhibiting the function of osteoclasts;^24^ it is therefore commonly used for the treatment of osteoporosis.^26^ Results from randomised controlled trials have shown that calcitonin stabilises and produces a short-term increase in BMD.^7-9^ In an 18-week follow-up histometric study on healthy rabbits, Januário et al^16^ reported that administration of salmon calcitonin improved bone mass following titanium implant insertion. Apostu et al^5^ reported that calcitonin improved BMD and had a positive impact on osseointegration.From a clinical perspective, there is one 24-month follow-up case-control study in which one of the osteoporotic patients with dental implants was treated with calcitonin.^13^ In this study,^13^ implant-related osseous complications were reported in none of the patients.

Platelets isolated from blood are an autologous source of growth factors. Platelet-rich fibrin (PRF) is an immune and platelet concentrate that facilitates healing and immunity.^28^ It consists of a fibrin matrix polymerized in a tetra molecular structure, with incorporation of circulating stem cells, leukocytes, cytokines, and platelets.^10^ The protocol of PRF preparation is simple and the armamentarium required is comparable to that of platelet-rich plasma.^28^ Results from a histomorphometric study on rabbits showed that local application of PRF at the implant osteotomy site provided faster osseointegration by increasing the rate and amount of new bone formation (NBF) during the early healing period around implants.^25^ Results from another recent study^23^ on rabbits showed that local application of PRF around implants with induced osseous defects augments NBF and increases BIC. It is hypothesised that systemic calcitonin delivery with adjunct local PRF therapy is more effective in augmenting osseointegration than calcitonin delivery alone under experimental osteoporosis conditions. The aim of the present scanning electron microscopy (SEM)-based experiment was to assess the effect of systemic calcitonin delivery with and without adjunct local PRF therapy on osseointegration in ovariectomised osteoporotic rabbits.

## Materials and Methods

### Ethics Statement

The present experimental study was evaluated and approved by the Ethics Committee for Animal Research at the Faculty of Medicine, Cairo University, Cairo, Egypt.

### Study Subjects

Thirty pathogen-free old adult New Zealand white female rabbits were used. The age ranged between 9 and 12 months and the weight from 2.5–3.5 kg. All animals were kept in individual cages under the same environment and conditions throughout the study. The rabbits were maintained on a 12 h dark/12 h light cycle at room temperature (20°C) with 58% humidity. All rabbits were acclimatised in these conditions for at least 7 days before the initiation of the present study.

### Establishment of Osteoporosis Model

All animals underwent bilateral ovariectomy by a trained operator according to the protocol described in the study by Wanderman et al^35^ and were fed a low-calcium diet (bran food) for 45 days. To ensure the occurrence of systemic bone mass loss, all rabbits received densitometric evaluation of BMD using dual-energy x-ray absorptiometry in the region of the tibia where the implants would be inserted. An osteoporosis model was considered to be effectively established when the average BMD decreased by 20%.^22,32^

### Randomisation, Grouping and Allocation Concealment

All osteoporotic rabbits (n = 30) were randomly divided into 3 groups with 10 animals per group. Randomisation was done using a computer-generated randomisation list (www.randomization.com) by an independent investigator who was involved neither in the selection of the animals nor in the surgical procedures (SSA). In group 1, no treatment was offered (control group). The other animals received intramuscular injections of calcitonin without (group 2) and with (group 3) local PRF delivery prior to implant placement.

### Platelet-rich Fibrin (PRF) Preparation for Group 3 (CT-PRF)

Preparation of PRF was done according to the protocol described by Januário et al.^16^ In brief, from all animals in group 3, articular blood was withdrawn from the femoral artery fusing a gauge 20 sterile needle connected to 10 ml syringe. Eight ml autogenous blood was collected and placed into a 10-ml glass tube in the absence of anticoagulants and immediately centrifuged at 3000 rpm for 10 min. Following centrifugation, the fibrin clot representing the middle segment was separated with tissue pliers, and compressed between moist cotton gauze through plasma layer draining.

### Surgical Procedure and Drug Administration

All surgical procedures were performed under complete sedation, which was accomplished via intramuscular injection of ketamine (Ketamine HCL injection USP, Rotexmedica; Trittau, Germany) and xylazine (Rompun, Bayer; Leverkusen, Germany), by a trained investigator who was blinded to the study groups. Under completely aseptic conditions, an incision was made in the medial side of the knee-joint skin. For adequate tibia platform exposure, the knee muscle and joint capsule were dissected. To mark the site of implant insertion, a round bur with profuse saline irrigation was used for gentle drilling at the center of the tibia platform. A titanium implant (Super Line Dentium Implant System, Dentium; Seoul, Korea), 3.4 mm in diameter and 8 mm in length, was inserted bilaterally in the tibia in each rabbit. All implants were placed at bone level using an insertion torque of 30-35 Ncm; a covering screw was then placed on each implant. Following the surgical procedure of implant insertion, an intramuscular injection of 3.5 IU/kg calcitonin (Miacalcic Ampoules, 50 IU synthetic salmon calcitonin, Novartis Pharma Stein; Cairo, Egypt) was administrated to rabbits in groups 2 and 3 on alternate days for 12 weeks. In group 3, PRF was applied into the osteotomy site immediately before implant insertion.

### Euthanasia

At 12 weeks postoperatively, all animals were sacrificed using 50 mg/kg intravenous injection of sodium pentobarbital. The operator who performed euthanasia was blinded to the study groups.

### Scanning Electron Microscope and Energy-Dispersive X-ray Spectroscopy

Tibial segments were dissected and subjected to SEM using a technique described by Hipp et al.^15^ Each implant specimen was dehydrated in an ascending alcohol series for 10 h, then embedded in methyl methacrylate without decalcification. Sections were made through the longitudinal axis of the implants directly after polymerization. A low-speed diamond wheel was used to cut the embedded tissue into 150-μm-thick sections. To obtain a consistent surface finish, the sections were sanded on an abrasive paper. All SEM-based investigations were performed by a trained investigator. The magnetron sputtering device (JEOL; Tokyo, Japan) was implemented to coat the implant with a layer of gold. This was followed by implant examination using a high-resolution field-emission scanning electron microscope (FE-SEM, JEOL 6300F) connected to a computer screen (IBM; Armonk, NY, USA). The gap between the implant and bone around all threads was measured in micrometers (μm) at 4000–6000X magnification and compared in the three groups throughout the implant length (cervical, middle and apical thirds of the implant body). Specimens that showed complete osseointegration were further subjected to energy-dispersive x-ray spectroscopy (EDX),^29^ which is an analytical SEM technique for analysing the elemental composition or chemically characterising specimens. It was applied at different areas throughout the bone-implant interface. EDX analysis were performed by one investigator who was blinded to the study groups. The bone-to-implant contact (BIC) was measured and reported in percentage as described in the study by Kızıldağ et al.^23^ In short, BIC was defined as the amount of the implant surface that directly attached to mineralised bone without the interposition of soft connective tissue.^23^

**Fig 1:**
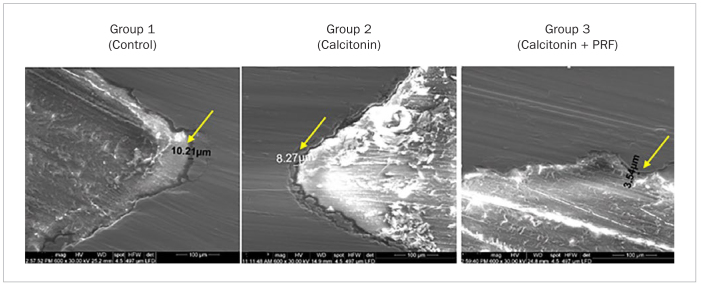
SEM images showing the gap widths (yellow arrows) in micrometers among the study groups.

### Statistical Analysis

Statistical analysis was performed using a software program (IBM SPSS Statistics Version 20 for Windows; Armonk, NY; USA). Data normality was assessed using the Kruskal-Wallis test, and gap widths were presented as means ± SD. Group comparisons were performed using one-way ANOVA and Bonferroni post-hoc adjustment tests. Tukey’s test was used for pairwise comparisons between the mean values when ANOVA test was statistically significant. The sample size calculation for a replication study was also performed based on the previous alpha value and type of distribution. The statistical power set at 0.90. Statistical significance was set at p < 0.05.

## Results

### Gap Width and BIC

Gap widths in the cervical (p < 0.001), middle (p < 0.001) and apical third (p < 0.001) were significantly higher in group 1 than in groups 2 and 3. Gap widths in the cervical (p < 0.001), middle (p < 0.001) and apical third (p < 0.001) were significantly higher in group 3 than in group 2 (Table 1). The mean BIC was statistically significantly higher in the cervical (p < 0.001), middle (p < 0.001) and apical thirds (p < 0.001) in group 2 compared with group 1 (Table 2). The mean BIC was statistically significantly higher in the cervical (p < 0.001), middle (p < 0.001) and apical thirds (p < 0.001) in group 3 than in groups 2 and 3 (Table 2).

**Table 1:** Comparison of mean ± SD of gap widths (μm) in the study groups

Gap width (μm)	Group 1 (control)	Group 2 (calcitonin)	Group 3 (calcitonin + PRF)
Apex	4.22 ± 0.51 μm*	0.86 ± 0.22 μm†	0.012 ± 0.006 μm
Middle	5.89 ± 0.47 μm*	1.86 ± 0.52 μm†	0.59 ± 0.06 μm
Cervical	7.83 ± 0.61 μm*	2.83 ± 0.64 μm†	1.29 ± 0.04 μm
Mean of the 3 sites	5.98 ± 0.74 μm*	1.85 ± 0.52 μm†	0.63 ± 0.005 μm

SD: standard deviation; PRF: platelet rich fibrin. *Compared with groups 2 (p < 0.001) and 3 (p < 0.001). †Compared with group 3 (p < 0.001).

**Table 2:** Comparison of mean bone-to-implant contact in the study groups

Bone to implant contact	Group 1 (Control)	Group 2 (Calcitonin)	Group 3 (Calcitonin + PRF)
Apex	20.26 ± 5.3%*	53.82 ± 6.4%†	92.81 ± 3.3%
Middle	23.56 ± 3.4%*	55.41 ± 5.3%†	94.11 ± 2.8%
Cervical	30.43± 2.4%*	60.28 ± 3.4%†	96.38 ± 2.3%
Mean of the 3 sites	21.76 ± 4.8%*	54.26 ± 4.1%†	95.68 ± 2.7%

PRF: platelet rich fibrin; *compared with groups 2 (p < 0.001) and 3 (p < 0.001); †compared with group 3 (p < 0.001).

### EDX Elemental Analysis of SEM and BIC

**Fig 2:**
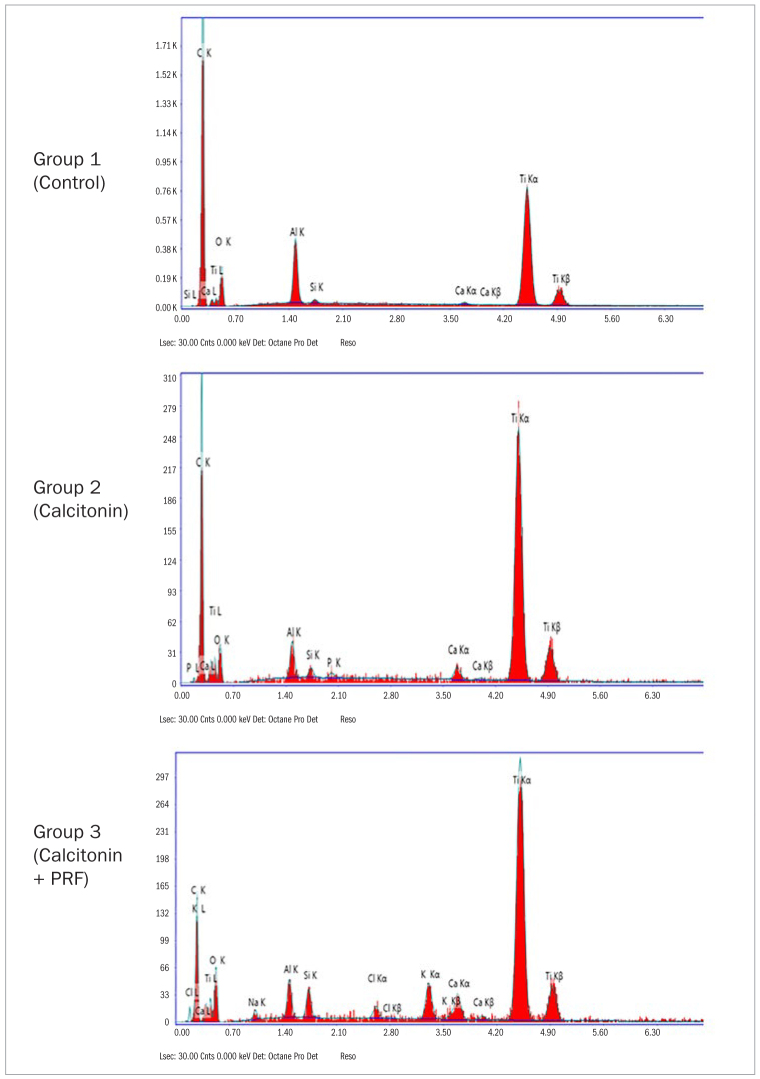
Energy-dispersive x-ray spectroscopy in the study groups.

The SEM results showed osseous tissues in varying degrees of ingrowth filling the grooves along the implant fixtures. SEM also showed minimal gap widths between the bone and implant surfaces in the cervical, middle and apical regions in group 3, in contrast to groups 1 and 2. Greater gap widths were detected between bone and implant surfaces in groups 1 and 2 than in group 3 (Fig 2). EDX elemental analysis detected bone mineralisation elements in addition to elemental titanium on the bone-titanium contact area (BIC), ensuring a maximum BIC in group 3 compared with groups 1 and 2 (Fig 2).

## Discussion

The present experimental study was based on the hypothesis that systemic calcitonin delivery with adjunct local PRF therapy is more effective in augmenting osseointegration than calcitonin delivery alone under experimental osteoporosis conditions. The present SEM findings are in accordance with this hypothesis, as gap widths between implants and bone were significantly higher in groups 1 and 2 (control and calcitonin injections, respectively) compared with group 3 (calcitonin + local PRF delivery). In addition, the BIC in the cervical, middle and apical thirds of group 3 implants was statistically significantly greater in group 3 compared with the other groups. To the authors’ knowledge from pertinent indexed literature, the present study is the first to report the effects of PRF on osseointegration in an experimental osteoporotic model. Traditionally, platelet-rich plasma (PRP) is used to induce new bond formation (NBF) around peri-implant osseous defects;^34^ the use of PRF is a more cost-effective and easier method than PRP for the induction of NBF.^28^ Moreover, PRF promotes hemostasis, promotes cell proliferation and migration, and facilitatesxxwhat? due to slow polymerization compared with PRP. These factors seem to have contributed to faster induction of NBF, thereby enhancing BIC in group 3 vs the other groups. According to Baeyenes et al,^6^ platelet concentrates (such as PRF in the present scenario) are also easy to use clinically and offer potential advantages such as bone regeneration and rapid wound healing. Those authors further proposed that platelet concentrates can be considered ‘new therapeutic adjuvants’.^6^ The authors of the present study support the proposal made by Baeyenes et al.^6^ Ideally, there should have been a fourth group in the present study in which local PRF application was used as the sole therapeutic regimen for inducing NBF. This could have helped clarify the potential role of PRF alone towards the closure of gaps between the bone and implant surfaces.

In an in vitro study, He et al^14^ assessed the effect of biological characteristics of PRF and PRP on the differentiation and proliferation of rat osteoblasts. The authors collected blood samples from young, healthy human adults (approximately 23 years old); PRP and PRF were extracted from the blood samples and quantified for transforming growth factor-beta (TGF-β) and platelet derived growth factor (PDGF). These PRF and PRP exudates were used to culture rat calvaria osteoblasts. The results showed that compared with PRP, PRF gradually released autologous growth factors and demonstrated a robust impact on the differentiation and proliferation of rat osteoblasts.^14^ The authors of the present study support the in vitro results by He et al.^14^ It is likely that the increased expression of growth factors, such as PDGF and TGF-β in the osteotomy sites in group 3 resulted in the osteoblastic activity, thereby enhancing NBF and BIC between implant threads and surrounding bone.

The main limitation of the present study is that the outcomes were based on results obtained from investigations on animal models. In the present study, the control group comprised animals that did not receive any form of treatment, whereas in the remaining the animals received calcitonin with or without adjunct PRF therapy. The authors speculate that an additional test group of animals that underwent PRF therapy alone would have strengthened the present study. It is hypothesised that treatment with PRF alone enhances NBF at peri-implant osseous defects. Further studies with additional relevant groups are therefore needed. From a clinical perspective, there are numerous local and systemic risk actors that jeopardise osseointegration by increasing peri-implant soft-tissue inflammation and crestal bone loss.^12^ A state of chronic hyperglycemia, which is a critical manifestation in patients with poorly-controlled diabetes and prediabetes has been directly associated with peri-implant mucositis and peri-implantitis.^1,4^ Moreover, it has also been reported that poorly-controlled diabetes contributes towards lower BMD. It is therefore speculated that the prevalence of a state of chronic hyperglycemia is more often manifested in patients with osteoporosis compared with systemically healthy individuals. This may in turn compromise osseointegration and the effect of PRF + calcitonin if used for the treatment of peri-implantitis in the former group. Tobacco smoking is a well-documented local risk factor for peri-implant diseases;^2,3^ habitual smoking has also been reported to compromise the outcomes of oral surgical interventions such as implant therapy.^18^ Furthermore, smoking is a risk factor for alveolar bone loss and compromised BMD.^19,31^ It is hypothesised that the NBF following calcitonin + PRF therapy around dental implants is compromised in smokers and diabetic patients with osteoporosis. It is also expected that histological assessment of peri-implant osseous tissues would demonstrate a significantly greater number of osteoprogenitor cells in group 3 than in groups 1 and 2. Based upon such limitations, it is too early to apply the present results to clinical settings. Further studies are needed on the hypotheses described above.

## Conclusion

When used as an adjunct to calcitonin, PRF enhances osseointegration in an experimental osteoporosis model. However, further well-designed studies with the inclusion of additional groups (treatment with PRF alone) are needed.
